# Synthesis of novel vanillin-amine hardeners fully derived from renewable bio feedstocks and their curing with epoxy resins to produce recyclable reprocessable vitrimers

**DOI:** 10.1016/j.heliyon.2023.e16062

**Published:** 2023-05-06

**Authors:** Muhammad Abdur Rashid, Md Nabiul Hasan, Md Abdullahil Kafi

**Affiliations:** Dhaka University of Engineering & Technology, Gazipur, 1707, Bangladesh

**Keywords:** Epoxy vitrimers, Vanillin imine hardener, Recyclable composite, Self-healing thermoset, Reprocess

## Abstract

Biobased epoxy vitrimers have reached intense interest in recent decades. The triggerable reverse bonds can be introduced into these crosslinked epoxy vitrimers through epoxy resins or hardeners. This study synthesized two imine hardeners, such as vanillin-butanediamine (V-BDA) and vanillin-hexanediamine (V-HDA), using biobased vanillin, butanediamine, and hexanediamine and their chemical structures were ensured by FTIR, ^1^HNMR, ^13^CNMR, and TOF-MS. The two novel hardeners were used to cure epoxy resins, rendering vitrimers with good reprocessability, self-healing, recyclability, and solvent resistance due to the reversible imine bonds. The flexural strengths and modulus of these cured resins were consistent with those of epoxy resins that were hardened with traditional amine-based hardeners. The cured resins maintained 100% of their *T*_*g*_ and flexural properties after being reprocessed up to three times. It was revealed that the cured epoxy vitrimers could be degraded entirely in a particular acidic solution capable of bond-exchanging reactions within 12 h at 50 ᵒC, allowing the thermoset matrix to be chemically recycled and the monomers regenerated. This versatile recyclability, combined with the use of fully biobased feedstocks to prepare the hardeners, provides an attractive approach to help achieve a sustainable circular composite economy.

## Graphical abstract

**Figure:**
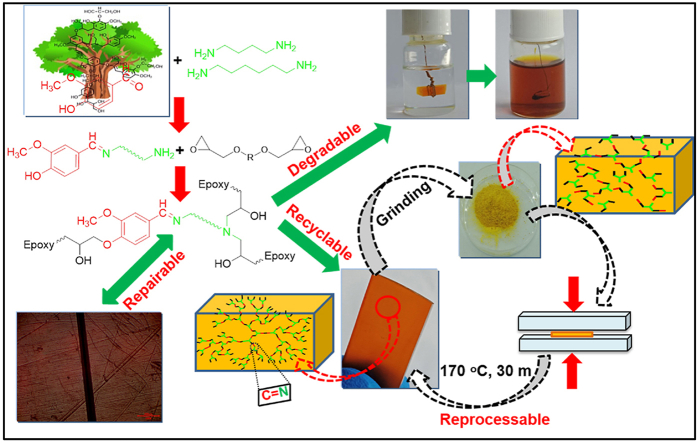

## 1 Introduction

Epoxy resins are thermoset and have balanced thermal, mechanical properties, chemical and dimensional stability, as well as versatile processability, allowing them to be extensively utilized in diverse fields of adhesives, castings, coatings, microelectronic encapsulating, printed circuit boards, and high-performance composites [[1], [2], [3], [4]]. Due to their high density of crosslinks, traditional epoxy resins cannot be reformed, mended, or recycled, similarly to thermoplastics [[5], [6], [7], [8]]. Therefore, waste materials from production and end-of-life parts, especially from large structure composite applications, such as wind blades and vehicles, usually end up in landfill or are pyrolyzed, causing environmental pollution [8]. A novel approach to producing reprocessable and recyclable thermoset polymers, called covalent adaptable networks (CANs), has been formed by incorporating dynamic covalent bonds. Such covalent linkages are capable of breaking or reforming in response to additional stimuli, such as stress, temperature, radiation, solvents etc., by performing reversible de-polymerization/dissociative processes or exchange/associative reactions [[9], [10], [11], [12]] and making the polymer thermosets reusable, deformable, reprocessable, weldable, rectifiable and soluable. Dissociative CANs go through dimensional changes via convertible de-polymerization or recreation of crosslinked structures; as a result, their mechanical properties also drastically change during reformation [[13], [14], [15]]. Contrarily, associative CANs or vitrimers maintain their network integrity and cross-link density without degradation or liquefication [5,[16], [17], [18], [19], [20], [21]]. In addition, vitrimers are made up of molecular, covalent structures that can bond exchange and change their topology through heat. It can flow as viscous liquids at high temperatures, but at low temperatures, bond-exchange interactions are impossible; hence vitrimers behave as traditional thermosets. The name vitrimer was adopted by Leibler's group in 2011 based on their study on reversible *trans*-esterification reactions [5]. After that, the research on vitrimers surged, covering dynamic bond exchanges such as disulfide [20,22], ester [10,21,23], imine [17,19,24], diels-alder adduct [13,15], acetal [[25], [26], [27]], spiroacetal [28,29] and siloxane [30]. Among these chemistries, the imine/Schiff base is particularly interesting for epoxy thermosets since it can exhibit several triggerable responses, including transamination, imine metathesis, and imine condensation, even without the addition of catalyst [31,32], and it yields excellent thermal, mechanical properties, processability, and reprocessability [19,24,33,34].

In addition to the recyclability rendered by the vitrimers, epoxy resins originating from sustainable biomaterials have an additional advantage in reducing fossil fuel use and carbon dioxide emission [3,16,19]. A number of recyclable thermosets prepared from bio/renewable resources have been reported [3,6,19]. However, these materials exhibited low thermal and mechanical performances, poor chemical and solvent resistance compared to conventional epoxy resins, limiting their industrial utilization. One of the most readily available bioresources is lignin, which consists of an aromatic monomer that can be introduced into performance polymers [2,25,35,36]. Unfortunately, epoxy resins collected directly from lignin usually possess higher molecular masses, lower curing abilities, and poor solubility, except for monoaromatic vanillin derived from lignin [[36], [37], [38], [39], [40]].

Therefore, this study focuses on an alternative approach by combining lignin-based vanillin with biobased amines to prepare hardeners for epoxy resins, leading to the design and synthesis of two novel biobased imine hardeners/curing agents. In this research, vanillin was obtained from the fermentation of lignin, butanediamine came from the disintegration of amino acids in organisms and the metabolism of *Escherichia coli* bacteria, and hexanediamine was derived from biomass fructose syrup. Furthermore, the curing response of these imine-containing hardeners, i.e. vanillin-butanediamine (V-BDA) and vanillin-hexanediamine (V-HDA) with a bisphenol-F epoxy resin was investigated. The cured resins were studied for their thermomechanical performances, chemical stability, recyclability, repairability/self-healing, and degradability. It was demonstrated that the amine molecular weight affected the curing kinetics, mechanical and thermal performances, and the cured resins' recyclability. The cured resins showed good reforming and self-healing ability under hot pressing, allowing their mechanical properties to achieve 100% recovery after up to three reprocesses. The cured resins also had excellent chemical resistance and were degradable in acidic solution, allowing monomers to reclaim.

## 2 Experimental section

### 2.1 Materials

Fermentation lignin-derived vanillin was obtained from Sigma-Aldrich Shanghai Trading Co. Ltd., China. Biomass butanediamine (BDA) and hexanediamine (HDA), 2,4,6-tri(dimethylaminoethyl)phenol (DMP-30) and others chemicals were collected from sinopharm chemical reagent co. ltd., Shangahi, China and used without further purify. Bisphenol-F epoxy resin (NPEF-170, EEW 170), and diglycidyl ether of neopentyl glycol (NPGDGE, EEW 138) were supplied by Zheijiang Baihe Advanced Composites Ltd., China.

### 2.2 Synthesis of vanillin-butanediamine hardener (V-BDA)

**Fig. 1:**
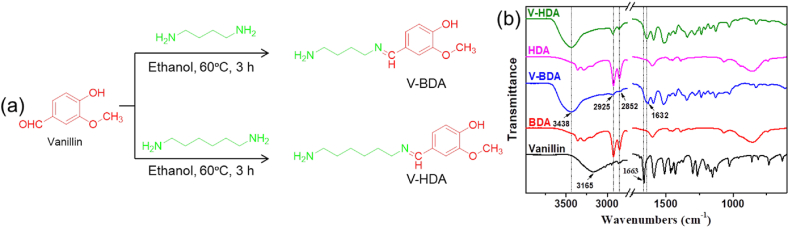
(a) Synthesis techniques of V-BDA and V-HDA from lignin-derived vanillin, and (b) FTIR spectra of vanillin, BDA, V-BDA, HDA, and V-HDA.

250 mL of anhydrous ethanol was used to dissolve 26.45 g (0.3 mol) of butanediamine before it was poured to a flask with a reflux condensation tube, magnetic stirrer, and thermometer. 15.21 g (0.1 mol) of vanillin was solubilized in 50 mL of ethanol before being poured dropwise to the flask and magnetically stirred for an hour at ambient temperature. The mixed solution was heated to 60 ᵒC and maintaining for 3 h under N_2_ purge. The synthesized component was put in a rotary evaporator to separate the solvent and remaining amount of butanediamine. A single solvent recrystallization method using ethyl acetate was used to purify the resulting component. The finished product was a light yellow powder (19.46 g) with a yield of 81%. The synthetic technique is demonstrated in Fig. 1a**.** HRMS (ESI^+^) *m*/*z*: [M+H]^+^ cal. 223.14, found 223.18; *m*/*z*: [M+Na]^+^ cal. 245.16 found 245.16. ^1^H NMR (400 MHz, DMSO‑*d*_6_, ppm) *δ*: 1.44 (m, 2H, –CH_2_-), 1.61 (m, 2H, –CH_2_-), 1.98 (s, 2H, –NH_2_), 2.64 (m, 2H, –CH_2_-), 3.51 (m, 2H, –CH_2_-), 3.75 (s, 3H, –CH_3_), 5.84 (s, 1H, –OH), 6.76 (d, 1H, Ar–H), 7.04 (d, 1H, Ar–H), 7.28 (s, 1H, Ar–H), 8.12 (s, 1H, –CH–). ^13^C NMR (400 MHz, DMSO‑*d*_6_, ppm) *δ*: 28.46, 28.59, 40.26, 55.35, 60.20, 109.65, 115.43, 123.06, 126.36, 148.49, 151.58, and 160.33.

### 2.3 Synthesis of vanillin-hexanediamine hardener (V-HDA)

The V-HDA hardener was synthesized and purified the same way except using hexanediamine, with a yield of 83.55%. The synthetic technique is demonstrated in Fig. 1(a)**.** HRMS (ESI^+^) *m*/*z*: [M+H]^+^ cal. 251.17, found 251.19; *m*/*z*: [M+Na]^+^ cal. 273.15 found 273.17. ^1^H NMR (400 MHz, DMSO‑*d*_6_, ppm) *δ*: 1.19 (m, 2H, –CH_2_-), 1.23 (m, 2H, –CH_2_-), 1.28 (m, 2H, –CH_2_-), 1.52 (m, 2H, –CH_2_-), 1.94 (s, 2H, –NH_2_), 2.45 (d, 2H, –CH_2_-), 3.42 (m, 2H, –CH_2_-), 3.71 (s, 3H, –CH_3_), 5.68 (s, 1H, –OH), 6.71 (d, 1H, Ar–H), 6.99 (d, 1H, Ar–H), 7.24 (s, 1H, Ar–H), 8.06 (s, 1H, –CH–). ^13^C NMR (400 MHz, DMSO‑*d*_6_, ppm) *δ*: 26.36, 26.81, 30.83, 32.29, 41.20, 55.48, 60.48, 109.81, 115.49, 123.01, 126.81, 148.46, 151.19, and 160.21.

### 2.4 Preparation of vanillin-amine hardeners based vitrimers

The above-synthesized imine hardener V-BDA (11.95 g) was dissolved in 25 mL of DCM and 25 mL of anhydrous ethanol (1:1 ratio) solvent mix. The solution was mixed with an epoxy resin comprising 60% DGEBF (15.0 g) and 40% NPGDGE (10 g) to maintain a low viscous mixture with an EEW value of 155 and 0.4 g of DMP-30 as a catalyst. The stoichiometric molar proportion of V-BDA to epoxy resin monomer was 1:3. Vigorous stirring for 10 min at normal temperature; the solvents were subsequently removed using a vacuum pump at 40 ᵒC. The mixture was quickly transferred into predetermined metal molds for different investigations and degassed at 50 ᵒC for 30 min. Lastly, the degassed epoxy polymer was heated at 80 ᵒC for 2 h, 130 ᵒC for 2 h, and 170 ᵒC for 2 h. The same method was applied to form a vanillin-amine hardener-based vitrimer cured by V-HDA.

### 2.5 Reprocessing of vanillin-amine hardeners based vitrimers

The vitrimer blocks were mechanically ground into a fine powder, charged into a metal mold, and compacted by a heat compression device at 170 ᵒC and 0.3 MPa for 0.5 h. The regenerated specimens were adequately removed from the metal mold after cooling to ambient temperature. Three cycles of reprocessing were performed.

### 2.6 Characterization

^1^H NMR and ^13^C NMR signals were investigated on a Bruker Avance (400 MHz) analyzer at normal temperature utilizing methyl sulfoxide-d6 (DMSO‑*d*_6_) as a deuterated solution to liquefy the specimens and tetramethylsilane (TMS) as the standard solution. The chemical shifts of ^1^H NMR and ^13^C NMR peaks are defined in ppm. Fourier-transform infrared (FT-IR) spectra were performed on a Thermo Nicolet 6700 FT-IR spectrometer through the KBr pellet system with the wavenumber limit of 400–4000 cm^−1^. An Agilent 7250 mass spectrometer was used to conduct high-resolution time-of-flight mass spectrometry (TOF-MS). The non-isothermal curing performances of the epoxy polymer were evaluated on a Polyma DSC 214 differential scanning calorimeter from 25 to 210 ᵒC at diverse heating ramps, including 5, 7.5, 10, 12.5, and 15 ᵒC/min under nitrogen gas. The thermal decomposition performance of the samples was evaluated by a TGA 4000 Thermogravimetric analyzer (TGA) (TA Instruments, USA) under nitrogen purge. The cured sample was heated in the limit of 50–700 ᵒC with a ramp rate of 10 ᵒC/min. The thermo-dynamic performances were tested using TA Instruments (DMA Q800, USA) with a ramp rate of 3 ᵒC min^−1^ and frequency of 1 Hz in the dual cantilever way. DMA was also used to perform stress relaxation in the tension mode. A rectangular sample (12.0 mm × 6.0 mm × 1.0 mm) was adjusted initially with 0.001 N force and then thermal equilibrium about 30 min for each experiment temperature. The specimens were stretched to 1% of their initial span and extended throughout the experiment. The relaxation modulus of the specimen was measured over time at a particular temperature until it reached equilibrium.

Three-point bending or flexural tests were carried on 203B-TS universal testing equipment (Wance, China) in accordance with ASTM D790 using a rectangle sample at a test speed of 1 mm/min at normal temperature. At least five reliable tests were used to calculate the strength.

## 3 Results and discussion

### 3.1 Characterization of the imine hardeners

As presented in Fig. 1a, the imine hardeners are formed via a reversible condensation between an aldehyde and a primary amine. FTIR, NMR, and TOF-MS spectroscopy characterized and confirmed their chemical structures. In the FTIR spectrum (Fig. 1b), the absorption peak of aldehyde groups (CHO) in vanillin is 1663 cm^−1^ and finally absent in the synthesized component. Additionally, a new peak at 1632 cm^−1^ exits owing to the creation of imine bonds [16,19]. The broadband about 3438 cm^−1^ can be allocated to the mutual absorptions of amine (N–H) and hydroxyl (O–H) stretching vibrations. The FT-IR spectra of V-BDA and V-HDA exhibit the same absorptions. The difference in absorptions due to different numbers of –CH_2_- in the diamines is normally reflected by the fingerprint regions, which are insignificant.

**Fig. 2:**
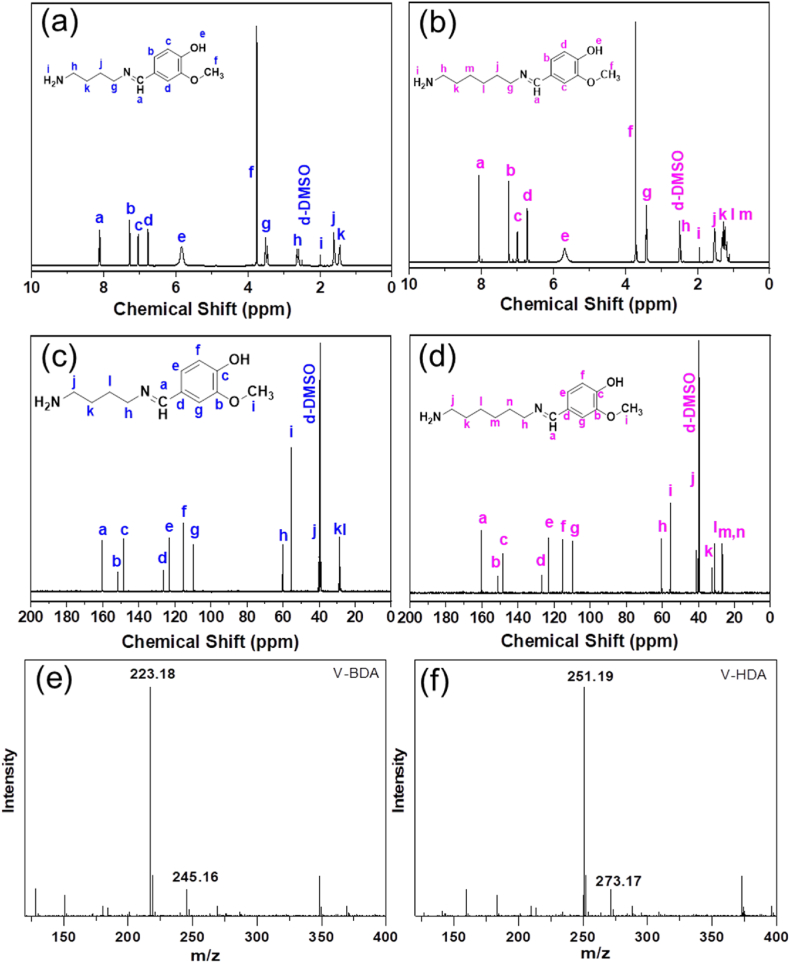
Proton and carbon NMR and mass spectra of V-BDA (a, c, e) and V-HDA (b, d, f).

^1^H NMR and ^13^C NMR further verified the structures of the imine hardeners. As presented in the ^1^H NMR spectra (Fig. 2a and b), the imine proton (H–C=N-) can be attributed to the absorption peak at 8.1 ppm, whereas the aldehyde proton (CHO, ∼9.5 ppm) in the precursor (i.e. vanillin) is absent. The proton in the hydroxyl group (-OH) is represented by the peak signal at ∼5.7 ppm, while the proton in the amino group (-NH_2_) is represented by the peak signal at 2.00 ppm. This suggests that only one of the biamine's amino groups combines with one of the vanillin's aldehyde groups, leaving the other amino group and the –OH group unaffected. ^13^C NMR in Fig. 2c and d shows the imine carbon (a) at 160 ppm, while the cabon of aldehyde (would be at 190 ppm) is not present as well, implying the creation of the proposed imine bond. In addition, the high-resolution mass spectrum displays the [M+H]^+^ ion at *m*/*z* = 223.18, and 251.19, respectively, which are consistent with the predicted structure of V-BDA and V-HAD (Fig. 2e and f). These findings support the effective synthesis of Schiff-based compounds from the interaction of vanillin and amine.

### 3.2 Curing reaction characteristics

**Fig. 3:**
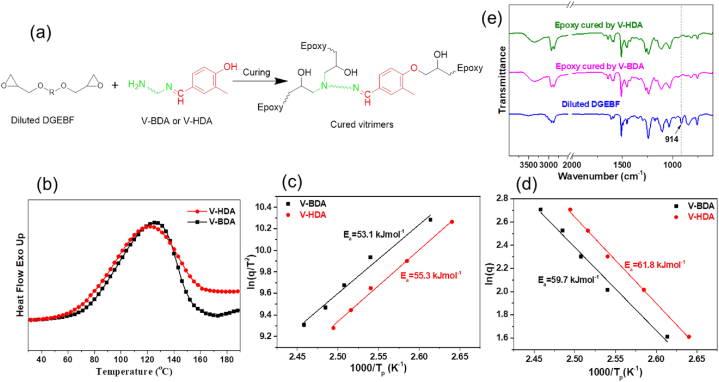
Schematically demonstration of the curing process of epoxy monomers and dynamic imine bond-containing curing agents (a); DSC curvess of epoxy and two imine curing agents (b); plots of –*ln(q/T*^*2*^*)* against *1/T*_*p*_ according to Kissinger's model (c); plots of *q* against *1/T*_*p*_ according to Ozawa's model (d); FT-IR spectra of diluted DGEBF, epoxy/V-BDA, and epoxy/V-HDA (e).

According to the V-BDA and V-HDA structures, the epoxy molecules can combine with both hydrogens on the amine and hydroxyl groups, as illustrated in Fig. 3a. However, the primary amine hydrogens react rapidly with the epoxy molecules, and hydroxyl hydrogens are much less reactive as well as typically need catalysts such as DMP-30 [41]. The DSC curves in Fig. 3b show that the two imine hardeners render similar curing characteristics, showing similar *T*_*peak*_*, T*_*onset,*_ and reaction enthalpy. The curing reaction energies (*E*_*a*_), calculated from two models, Kissinger's [42] and Ozawa's [43] models, can further reveal the curing mechanisms. The Kissinger model is presented by Equation (1):

(1) $$-\ln\left(\frac{q}{{T_{p}}^{2}}\right)=\frac{E_{a}}{RT}-\ln\left(\frac{AR}{E_{a}}\right)$$

where *q* and *T*_*p*_ denote the ramp rate and highest temperature, respectively; *E*_*a*_, *R,* and *A* denote the apparent curing reaction energy in kJ/mol, gas constant (8.314 J/mol.K), and the pre-exponential factor, correspondingly. The curing energy can be estimated from *–ln(q/T*_*p*_^*2*^*)* versus *1/T*_*p*_ curves [42]. Ozawa's model is represented by Equation (2):

(2) $$lnq=-1.052\frac{E_{a}}{{RT}_{p}}+\ln\left(\frac{{AE}_{a}}{R}\right)-lnF\left(x\right)-5.331$$

where *F(x)* is a conversion-dependent term. Hence, the slope of a linear fitting of ln *q* versus 1/*T*_*p*_ is utilized to decide the activation energy (*E*_*a*_) [43].

Fig. 3c and d presents the linear fits of both models, and the calculated *E*_*a*_ are recorded in Table S1. *E*_*a*_ in Table S1 confirms that both hardeners behave the same in reacting with epoxy, and the longer aliphatic chain in V-HDA had little effect on reactivity. In addition, these *E*_*a*_ values (53–62 kJ/mol) are consistent with those of traditional amine/epoxy systems (50–70 kJ/mol) [16,44]. It confirms that the curing reactions of both imine hardeners are acceptable for practical applications. Further, the vitrimers cured by imine hardeners were examined by FTIR (Fig. 3e). The spectra show no absorption peak by the epoxy molecules at 914 cm^−1^. The broad hydroxyl band at 3440 cm^−1^ indicates the formation of hydroxyl groups (OH) from the epoxide-opening response of the epoxy molecule.

### 3.3 Thermo-mechanical performance of imine-hardeners cured vitrimers

**Fig. 4:**
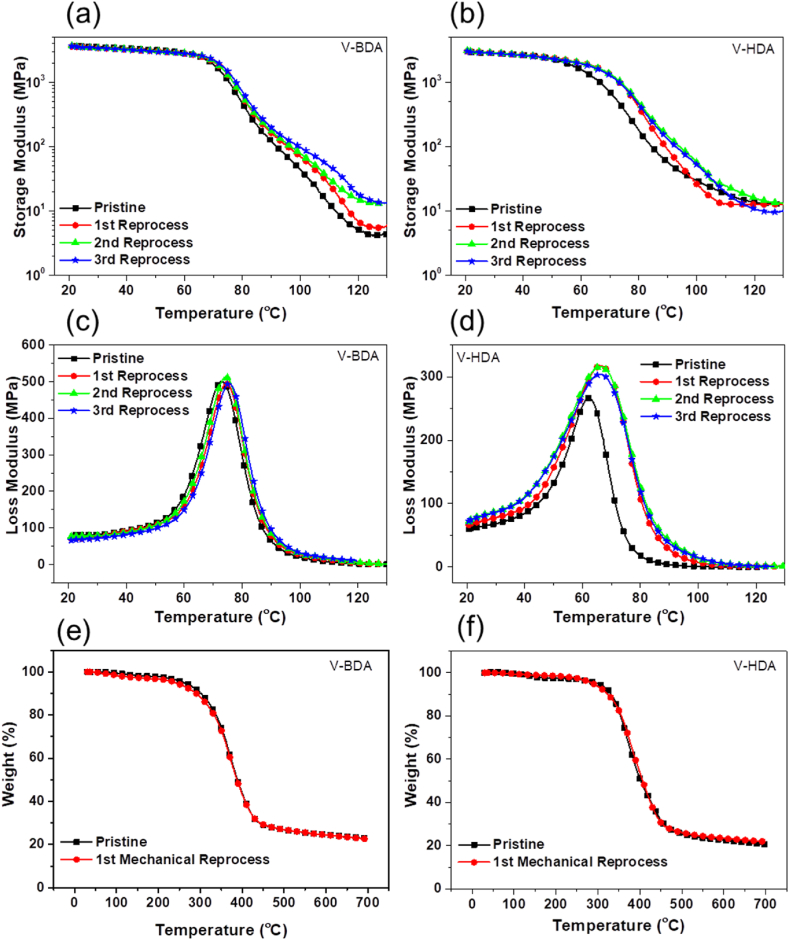
Storage and loss modulus vs. temperature plots of the pristine and reprocessed epoxy vitrimers cured by V-BDA (a, c) and V-HDA (b, d); TGA profiles of the pristine and reprocessed vitrimers cured by V-BDA (e) and V-HDA (f).

The thermo-mechanical performances of the pristine and reprocessed vitrimers cured by V-BDA and V-HDA were tested by DMA, as displayed in Fig. 4a–d and Table 1. The peak temperature of the loss modulus is reported as the glass transition temperature (*T*_*g*_). The first observation is that the epoxy resin, either pristine or reprocessed, that was cured with V-BDA had significantly higher storage modulus and *T*_*g*_ (3617 MPa and 75 ᵒC) than those of resins cured with V-HDA (2872 MPa and 66 ᵒC). This effect is evidently the result of differences in the aliphatic chain lengths in the diamines, with HDA having two more methylene groups than BDA. The second observation is that the epoxy resins cured with both hardeners exhibit good reprocessability, as demonstrated by the consistent storage modulus and *T*_*g*_ of both the pristine and reprocessed resins, even after being reprocessed three times due to the effective imine metathesis.

**Table 1:** DMA and mechanical results of the pristine and reprocessed epoxy vitrimer.

Samples	Storage modulus @25 °C (MPa)	*T*_*g*_ (⁰C)	*F. S. (MPa)	*F. M. (MPa)
Pristine epoxy/V-BDA	3617	74	88.4	3169
1st reprocessed epoxy/V-BDA	3527	75	95.9	3182
2nd reprocessed epoxy/V-BDA	3511	75	100.8	3255
3rd reprocessed epoxy/V-BDA	3476	75	102.1	3392
Pristine epoxy/V-HDA	2872	62	83.0	2242
1st reprocessed epoxy/V-HDA	2974	66	87.0	2333
2nd reprocessed epoxy/V-HDA	2980	66	89.2	2551
3rd reprocessed epoxy/V-HDA	2901	66	92.2	2514

[*F.S. = Flexural strength; *F.M. = Flexural modulus].

The thermo-stability of the cured vitrimers was tested using TGA, as presented in Fig. 4e and f and Table S2, and represented by the degradation temperature for 5% weight loss (*T*_*d5*%_), the temperature at the fastest weight loss (*T*_*dmax*_) and the residual weight percent at 700 °C (*Char*_*700*_). Fig. 4(e) and (f) show that one-stage degradation is apparent for all cured resins, pristine or reprocessed. Again, the thermal stability of epoxy vitrimers cured by these two imine hardeners is not significantly different. However, the initial degradation of V-BDA is lower than that of V-HDA because of its lower aliphatic chain structure. Furthermore, the degradation and thermal stability are not different compared to epoxy resins cured with conventional amine hardeners [45].

### 3.4 Mechanical performance of imine-hardeners cured vitrimers

**Fig. 5:**
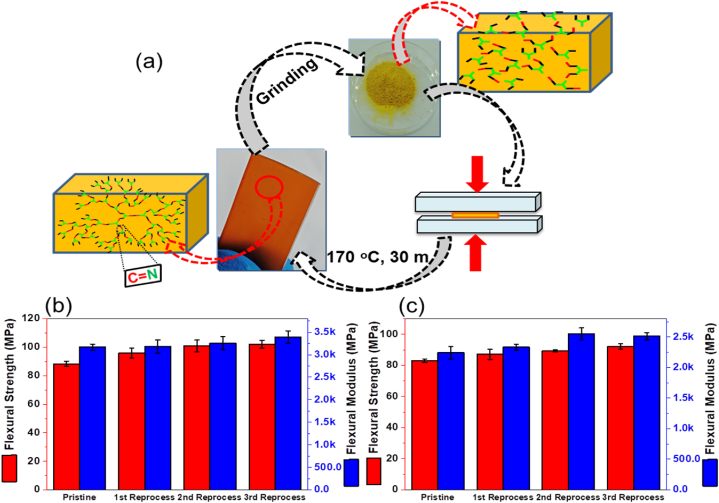
(a) Schematic diagram of thermally recyclable vitrimers cured by imine hardeners; 3-point bending or flexural strength and moduli of the virgin and reprocessed epoxy vitrimers cured by V-BDA (b) and V-HDA (c).

The 3-point bending or flexural strengths of the epoxy vitrimers cured by V-BDA and V-HDA hardeners were tested, and the relevant results are presented in Fig. 5b, c and Table 1. Not surprisingly, the defect-dominated flexural strengths of epoxy resins cured by V-BDA (88 MPa) are close to those cured by V-HDA because of their very similar cure thermodynamic and reaction kinetic properties. Likewise, their significantly different moduli (3169 and 2242 MPa, respectively) reflect the differences in *T*_*g*_ and crosslinking density, as expected for a long chain with HDA. Because imine bonds are capable of dynamic exchange, giving crosslinked epoxy resins vitreous properties [17,19,24], reprocessing becomes feasible as the network changes its topology without viscosity reduction and loss of materials integrity. The reprocessing of epoxy resins cured with both imine hardeners was conducted according to the process described in the experimental section and Fig. 5a. As revealed by the data in Fig. 5b, c and Table 1, the 3-point bending or flexural strengths of the reprocessed samples increase, indicating effective dynamic bond exchange with the possibility of increased cross-linking density. Furthermore, the increase in flexural modulus in the cases of both hardeners evidently implies that the degree of crosslinking increased as well, each time the cured resin was reprocessed, possibly due to the post-cure effect. Several studies reported making comparable observations [44,46,47]. Notably, the flexural characteristics of epoxy vitrimers cured by both imine hardeners, either pristine or reprocessed up to three times, are consistent with those of the corresponding epoxy resins cured by traditional amine hardeners [24,48].

### 3.5 Dynamic performance of imine-hardeners cured vitrimers

**Fig. 6:**
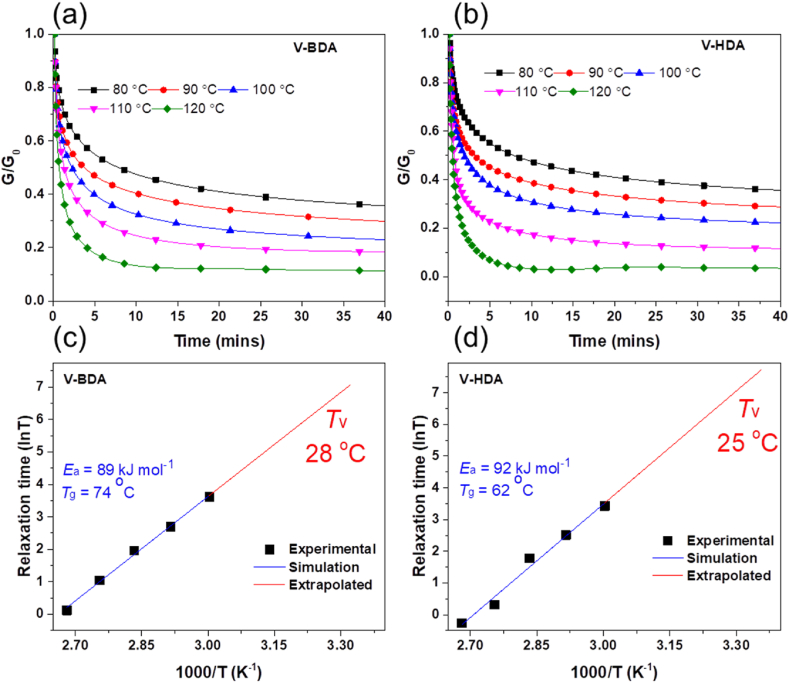
Normalized stress relaxation profiles (a, b) and fitting lines of relaxation time (ln *τ*) against *1000/T* (c, d) of vitrimers cured by V-BDA and V-HAD.

The dynamical performances of vitrimers treated by both imine hardeners (V-BDA and V-HDA) were examined using stress relaxation experiments. Their crosslinked networks can be continuously rearranged owing to the heating-driven retrograde imine metathesis, leading to stress relaxation of the curable vitrimers [32]. Fig. 6a and b displays the time-dependent normalized relaxation modulus (G/G_o_) of vitrimers cured by both imine hardeners over the temperature range of 80–120 ᵒC. Based on the viscoelastic model of Maxwell, the typical relaxation time (τ) of curable vitrimer is denoted as the relaxation modulus of 1/e (63.3%) of the starting modulus [49]. As expected, the τ values of epoxy vitrimers cured by both imine hardeners decline with raising the heating rate owing to the increased reversibility of imine linkages. The Arrhenius relationship (Equation (3)) can be applied to define the value of τ and test temperature (*T*).

(3) $$\tau \left(T\right)=\tau_{0}\;\exp\left(\frac{E_{a}}{RT}\right)$$

where *τ*_*0*_*, R,* and *E*_*a*_ represent the specific relaxation time at boundless temperature, ideal gas constant, and activation energy, correspondingly. Fig. 6c and d shows the relevant result of ln *(τ)* versus *1000/T* for the vitrimers treated by both imine hardeners, and the slope of the fitted line can be used to calculate the *E*_*a*_ values. The *E*_*a*_ values of vitrimers treated by V-BDA and V-HDA are 89 and 92 kJmol^-1^, respectively. These values are in the range of vitrimers with dynamic imine bonds recently published (*E*_*a*_ = 33.5−129 kJ/mol) [19,24]. The *E*_*a*_ values represent that both hardeners behave similarly in stress relaxation and that the longer aliphatic chain in V-HDA had no effect. In addition, these lower *E*_*a*_ values indicated that the dynamic imine exchange processes in these resins did not necessitate as much heat, making them more suited for repairing and reprocessing.

Another notable feature of triggerable chemistry is the topological freezing transition temperature (*T*_*v*_), which is specified as the temperature when the epoxy vitrimers maintain a viscosity of 10^12^ Pa s [5]. Thermodynamically, the interexchange ratio between reversible networks becomes extremely sluggish during the heat goes lower *T*_*v*_. Equation (4) can be used to get the result for τ in *T*_*v*_ [49].

(4) $$\eta =\frac{E^{´}\tau }{3}$$

where *η* and *E′* signify the viscosity at *T*_*v*_ (10^12^ Pa s) and the equilibrium rubbery plateau modulus of vitrimers, correspondingly. Then, *T*_*v*_ values were calculated using the Arrhenius fitted lines of ln *τ* (at *T*_*v*_), as illustrated in Fig. 6c and d. These results of epoxy vitrimers cured by V-BDA and V-HDA are 28 ᵒC and 25 ᵒC, respectively, significantly lower than their *T*_*g*_ values (62–74 ᵒC). Notably, the reversible imine metathesis in the vitrimers structures is also influenced by polymer backbone chain flexibility (related to *T*_*g*_). The bond exchanging mechanisms become locked during the cured vitrimer reaches a vitreous form. Thus, the stress relaxation of such epoxy vitrimers is dramatically accelerated as the temperature increases over their *T*_*g,*_ allowing them to perform effective repairing and recycling functions.

### 3.6 Self-healing performance

**Fig. 7:**
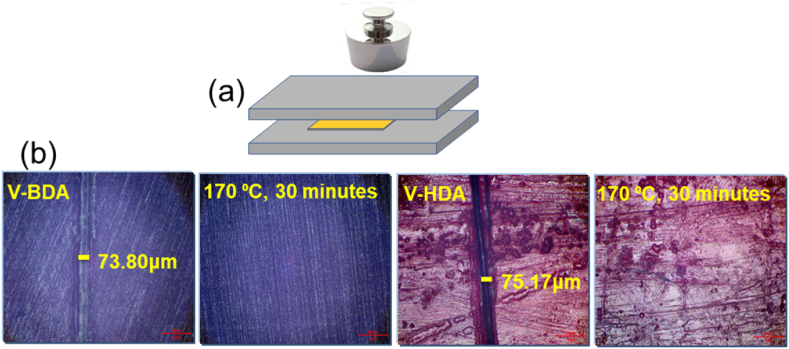
Demonstration of self-healing and repairing cured epoxy vitrimers: a) specimen heat treatment schematic; b) optical micrographs of cured epoxy vitrimers before and after treatment.

Epoxy resins applied to structural materials are generally brittle owing to their high degree of crosslinking and are susceptible to micro or macrocracks. Normal thermosets are also non-repairable. Vitrimers may undergo self-healing when conditions are favorable for bond exchanges, making this polymer particularly interesting for applications in structural materials. To examine the healing or mending performance, the cured epoxy resin blocks had their surfaces cut and were placed between two steel plates, shown in Fig. 7a, and held at 170 ᵒC for 30 min in a traditional oven. An optical microscope was utilized to examine the changes in the cut widths before and after the treatment, presented in Fig. 7b. It is apparent that the cuts in both resins were removed, and smooth surfaces were regenerated after the treatment, demonstrating effective vitrimeric performances.

### 3.7 Recycling of cured vitrimer by chemical degradation

**Fig. 8:**
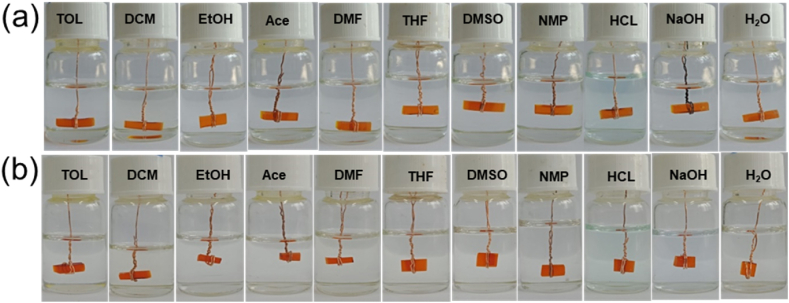
Photograph of cured EP-V-BDA (a) and EP-V-HDA (b) after 48 h in various solvents at room temperature.

Solvent resistance is a significant property that differentiates thermosets from thermoplastics. Vitrimers are thermoset polymeric networks and, therefore, should resist solvents such as normal thermosets. Hence, the epoxy resins cured by the two novel imine curing agents were subject to dissolution test using common and representative solvents, including water (H_2_O), NaOH (1 M), HCl (1 M), N-methyl-2-pyrrolidone (NMP), dimethylsulfoxide (DMSO), tetrahydrofuran (THF), dimethylformamide (DMF), acetone (Ace), ethanol (EtOH), dichloromethane (DCM), and toluene (TOL). A cured resin block of approximately 10 × 5 × 2 mm^3^ was immersed in solvents for 48 h at ambient temperatures, and its physical state was recorded and photographed, as shown in Fig. 8a and b. It is evident that none of the resin blocks dissolved in the tested solvents. This result agrees with similar observations published by researchers such as Memon and Zhao [19,24].

**Fig. 9:**
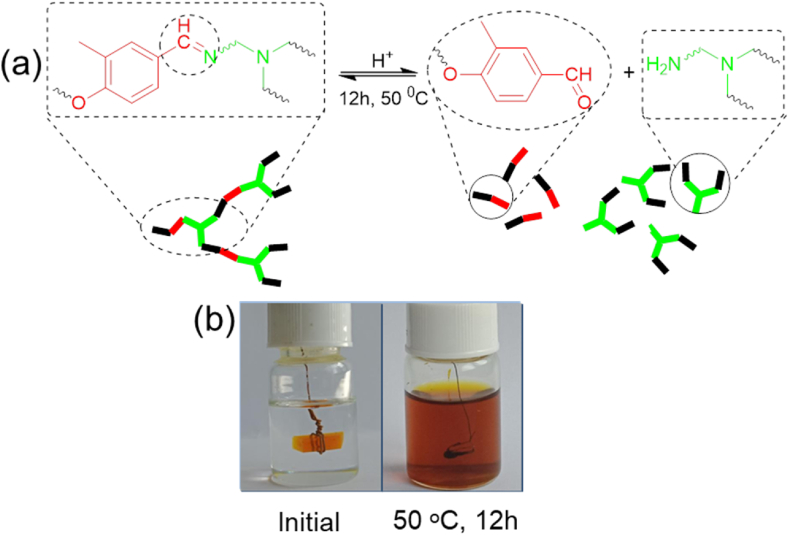
a) Degradation mechanism of imine linkage and b) photographs of epoxy vitrimers cured by imine hardeners (V-BDA) before and after degradation.

However, the bond exchange chemistries employed by vitrimers allow the crosslinked polymeric networks to be broken down and the thermoset materials to be degraded to chemically recycle the composite parts and/or reclaim the monomers. This is not viable for conventional composites with an epoxy resin matrix. Specifically, hydrolysis, transamination (amine-imine exchange), and imine metathesis (imine-imine exchange) reactions can be utilized to degrade epoxy resins cured with imine hardeners and recycle the amine and aldehyde monomers produced by the process [31,32], as schematically illustrated in Fig. 9a. In this work, the cured epoxy resins were fully degraded and dissolved within 12 h at 50 ᵒC under acidic solutions of 1 M H_2_SO_4_ in water/THF solvent mix (v/v = 2:8), as presented in Fig. 9b. Other scholars have made similar observations [17,19,24,50,51]. This reveals that while the two imines cured resins provide outstanding chemical stability in acidic and other solvent solutions at ambient temperature, they can also be chemically degraded and dissolved by raising their temperature in an acidic solution.

## 4 Conclusion

Based on the imine dynamic exchange covalent bond chemistry, two novel imine hardeners, V-BDA and V-HDA, were synthesized from reacting lignin-based vanillin with biobased diamines, butanediamine, and hexanediamine. These hardeners' chemical compositions and structures were characterized and confirmed by FTIR, ^1^HNMR, ^13^CNMR, and TOF-MS. Their curing dynamics, investigated by DSC, were similar to those of conventional amine hardeners. The DMA results indicated that the *T*_*g*_ of the cured resins is a function of the aliphatic chain length in the amine, wherein the butanediamine gave higher *T*_*g*_ than hexanediamine. The longer aliphatic chain also yielded the cured resin with a lower flexural modulus by lowering the crosslinking density. These novel imine hardeners endowed their cured epoxy resins with good vitreous polymer performances by exhibiting consistent *T*_*g*_ and flexural properties after reprocessing up to three times. Excellent self-healing characteristics were also demonstrated. By using a suitable solvent at a mild temperature of 50 ᵒC, the cured resins could be conveniently degraded, and the amine and aldehyde monomers are retrieved, giving the composite parts an alternative way to be recycled, in addition to being reprocessed like thermoplastics. This versatile ability for recycling, combined with using fully biobased feedstocks for hardeners, provides an attractive approach to help achieve a sustainable circular composite economy.

## Author contribution statement

Muhammad Abdur Rashid: Conceived and designed the experiments; Performed the experiments; Analyzed and interpreted the data; Contributed reagents, materials, analysis tools or data; Wrote the paper.

Md. Nabiul Hasan: Performed the experiments; Analyzed and interpreted the data; Wrote the paper.

Md. Abdullahil Kafi: Analyzed and interpreted the data; Wrote the paper.

## Data availability statement

Data will be made available on request.

## Declaration of competing interest

The authors declare that they have no known competing financial interests or personal relationships that could have appeared to influence the work reported in this paper.
