# Supplementary material for: Synthesis of novel vanillin-amine hardeners fully derived from renewable bio feedstocks and their curing with epoxy resins to produce recyclable reprocessable vitrimers
Source: Heliyon. 2023 May 6;9(5):e16062. doi: 10.1016/j.heliyon.2023.e16062 (PMC10196524; doi:10.1016/j.heliyon.2023.e16062)
Supplement: Supporting Information [file mmc1.docx]

**Supporting Information:**

**Synthesis of novel vanillin-amine hardeners fully derived from renewable bio feedstocks and their curing with epoxy resins to produce recyclable reprocessable vitrimers**

**Tanδ curves of V-BDA and V-HDA:**

**
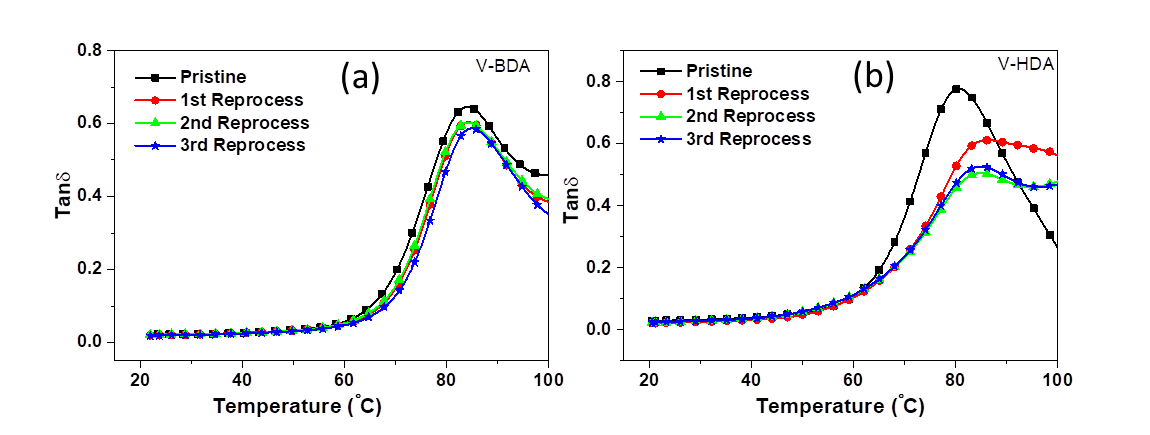
**

**Figure S1: Tanδ curves of V-BDA (a) and V-HDA (b).**

**Table S1**: Curing reaction activation energy (*E_a_*) of epoxy-hardeners from DSC

| Sample | *E_a_* (kJ/mol) | |
| --- | --- | --- |
|  | Kissinger | Ozawa |
| Epoxy/V-BDA | 53.1 | 59.7 |
| Epoxy/V-HDA | 55.3 | 61.8 |

**Table S2: TGA results of the pristine and reprocessed epoxy vitrimers**

| **Sample** | ***T_d5_*(ᵒC)** | ***T_dmax_*** | ***Char_700_* (%)** |
| --- | --- | --- | --- |
| Pristine EP/V-BDA | 260 | 365 | 23 |
| 1^st^ Reprocess EP/V-BDA | 250 | 370 | 23 |
| Pristine EP/V-HDA | 300 | 378 | 21 |
| 1^st^ Reprocess EP/V-HDA | 288 | 378 | 22 |
